# Scapula behavior associates with fast sprinting in first accelerated running

**DOI:** 10.1186/s40064-016-2291-5

**Published:** 2016-05-20

**Authors:** Mitsuo Otsuka, Taisuke Ito, Toyoyuki Honjo, Tadao Isaka

**Affiliations:** Faculty of Sport and Health Science, Ritsumeikan University, 1-1-1, Nojihigashi, Kusatsu-shi, Shiga 525-8577 Japan; PT Medical Center, 4-10-3, Roppongi, Minato-ku, Tokyo, 106-0032 Japan; Department of Mechanical Systems Engineering, National Defense Academy School of Systems Engineering, 1-10-20 Hashirimizu, Yokosuka, Kanagawa 239-8686 Japan

**Keywords:** 3D analysis, Acceleration, Biomechanics

## Abstract

The arm-swing motion is important for coordinated lower limb movement during a fast sprint and is composed of three-dimensional scapulothoracic and glenohumeral joint motion. Here, we aimed to clarify the role of the scapula during the initiation of a sprint running when sprinter run with high horizontal acceleration. Ten sports-active students participated in four 5-m dashes, with scapular constraint using non-elastic therapy tape (constraint condition) and without scapular constraint (free condition). The sprinting kinematics was assessed by a 16-camera motion capture system. In the constraint condition, the 2-m sprint time was significantly longer than that in the free condition. At the instants of foot-contact and take-off during the first step, no significant difference in the humerothoracic flexion angle was seen between these two conditions. In contrast, at the instants of foot-contact and take-off during the first step, the humerothoracic extension angle in the constraint condition was significantly smaller than that in the free condition. The forward leaning vector angle of center of mass during the first step was significantly greater than that in the constraint condition. Although no significant difference in hip extension and foot forward leaning angles was seen at the instant of foot contact during the first step between the two conditions, at the instant of take-off, the hip extension and foot forward leaning angles in the constraint condition were significantly smaller than those in the free condition. Therefore, scapular behavior in first accelerated running contributes to larger upper- and lower-limb motions and facilitates coordinating whole-body balance for a fast sprint.

## Background

For a fast sprint running, upper and lower limb movements have to be coordinated (Lockie et al. 2003). Running speed is reduced when the arm-swing motion is constrained, such as when holding a rugby ball (Grant et al. 2003) or a field hockey stick (Wdowski and Gittoes 2013). During jogging, the momentum at horizontal component of the upper arm, forearm and hand (we define these three segments as “arm”) is not directly associated with that of the whole-body center of mass (COM) because the backward- and forward-swinging motions of the arms are in opposite directions and the momentum is cancelled (Hinrichs 1987; Hinrichs et al. 1987). In contrast, in first accelerated running, the whole-body COM leans forward from the foot contact position (Kugler and Janshen 2010), suggesting that the relative momentum at horizontal component of both arms is not cancelled. Momentum is an indication of changes in velocity; therefore, the arm-swing motion in first accelerated running would contribute to generation of the sprint speed as well as plays a role in balancing the opposing extension and flexion motions of the lower limbs (Mann 1981; Slawinski et al. 2010b). In fact, in first accelerated running, the arm-swing motion affects lower limb kinematics (Lockie et al. 2014) and relates to the performance level of the sprinter in the 100-m dash (Slawinski et al. 2010a) and the sprint time (Lockie et al. 2003).

Humeral motion relative to the thorax is contributed by scapular motion relative to the thorax and humeral motion relative to the scapula (joints that relate to these motions are defined as humerothoracic, scapulothoracic, and glenohumeral joints, respectively (Codman 1934). Humerothoracic flexion and extension are associated with arm-swing motion in accelerated running, and this may be comprised of three-dimensional (3D) motion of the scapulothoracic and glenohumeral joints. The range of motion (ROM) of the scapulothoracic joint is half that of the glenohumeral joint during humerothoracic elevation (Inman et al. 1994), and the ROM of the scapulothoracic joint increases according to the movement velocity of the humerothoracic joint (Fayad et al. 2006). In first accelerated running, the flexion and extension angular velocity of the humerothoracic joint is high (approximately 700°/s) (Slawinski et al. 2010b), indicating that the ROM of the scapulothoracic joint is important during sprint running to enhance the ROM of the humerothoracic joint and the sprint speed. Therefore, the scapular behavior would be important for fast sprinting in first accelerated running.

Field sports such as football, lacrosse and field hockey require the skill to generate sprint speed quickly from a static or near static position (Sayers 2000). Sprint running is a fundamental skill required in most sports (Reilly et al. 2000; Sayers 2000). Therefore, clarifying the role of the scapula in first accelerated running will be useful for the players and coaches of many sports. The purpose of this study was to clarify the role of the scapula in sports-active students in first accelerated running. We hypothesized that when scapular motion is constrained, the arm-swing motion would be reduced, which changes the lower limb behavior and reduces the sprint speed.

## Methods

### Experimental approach to the problem

No previous studies have clarified the role of the scapula in sprint running. Noninvasively, mechanical and physiologic roles of a partial body position can be examined according to acute reduction by using joint taping (Koyama et al. 2014), a gripping tool (Grant et al. 2003; Wdowski and Gittoes 2013), or hypothermic anesthesia (Meyer et al. 2004). Many polyarticular muscles are attached around the shoulder joint, which is proximally located relative to the hand and foot. Therefore, based on the available methods, scapulothoracic joint taping can be considered the best valid method to restrict scapular behavior of the runner, and we compared the kinematics in first accelerated running between the scapula constrained condition and normal condition.

### Participants

Ten sports-active male students participated in this study [mean ± standard deviation (SD), age: 21.3 ± 1.1 years; height: 176.2 ± 6.0 cm; body mass: 69.3 ± 9.4 kg]. Each participant practiced daily physical activities that involved sprint running, including soccer (n = 5), rugby (n = 2), basketball (n = 2), and tennis (n = 1). The experimental protocol was approved by the Research Ethics Committee involving Living Human Participants at Ritsumeikan University (BKC-human-2013-12). All participants gave informed written consent before participation.

### Experimental procedure

The participants performed four 5-m dashes without (free condition) and with the scapula constrained (constraint condition). The participants sprinted from the same comfortable standing position with maximal effort in each trial. The order of the experimental trials in the free and constraint conditions was randomized. The experimental trials were performed in the laboratory room after a sufficient warm-up of 10 min including several submaximal dashes.

**Fig. 1:**
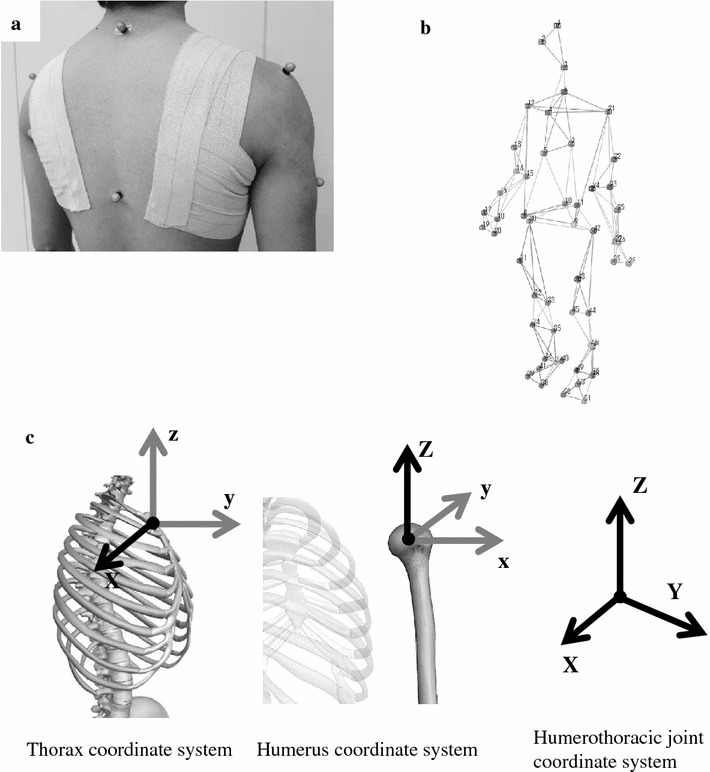
**a** Constraint condition created by taping both scapulae using non-elastic therapy tape. **b** Whole-body marker set. **c** Definition of center of rotation of segments and axes. In humerothoracic joint coordinate system, the X-axis was the X-axis of the thorax, the Z-axis was the Z-axis of the humerus, and the Y-axis was the common line perpendicular to the X- and Z-axis

A physiotherapist created the constraint condition by taping both scapulae symmetrically using non-elastic therapy tape (Fig. 1a). Before the experimental dash, humerothoracic flexion and extension functional tests were conducted separately, and each level of compression of both scapulae was assessed. In this humerothoracic flexion and extension functional tests, the participants were instructed to start from a comfortable standing position and perform full active flexion and extension in the sagittal plane (0–3 s), and then reverse the motion to return to the starting position (3–6 s) (Janes et al. 2012). The motion speed of these two functional tests was defined by the physiotherapist’s verbal instructions. We confirmed that the humerothoracic extension and flexion ROMs, whose motion is primarily used in arm-swing motion during running (Slawinski et al. 2010b), were restricted by taping both scapulae in humerothoracic flexion (ROM: 133.1° ± 4.3°–110.3° ± 9.1°, *P* < 0.05; *d* = 3.20) and extension (ROM: 50.3° ± 8.3°–27.7° ± 8.4°; *P* < 0.05; *d* = 2.71) functional tests.

### Data collection

Humerothoracic flexion and extension functional tests and sprinting kinematics were assessed by a 16-camera motion capture system (Raptor-E digital; Motion Analysis Corporation, Santa Rosa, CA, USA). The capture volume was 3.0 m × 2.0 m × 2.5 m (length × width × height). The 53 retro-reflective markers (9-mm diameter) were attached to the respective anatomical position of the whole-body based on modified marker set used by Slawinski et al. (2010a, b) (Fig. 1b). The validity of this method has been studied (Janes et al. 2012), and the measurement errors are estimated to be at a few degrees, which is similar to the magnetic sensor method (Lempereur et al. 2014). The 3D displacement data of the reflective markers was collected and sampled at 250 Hz. The ground reaction force (GRF) data was recorded at 1250 Hz using a force plate (TF-4060; Tec Gihan Corp., Kyoto, Japan) and was synchronized with the motion capture system by an analog/digital board.

### Data processing

The data of the 53 markers for each participant were filtered using a low-pass fourth-order, zero-lag Butterworth filter with a cut-off frequency of 6 Hz. A 15-segment rigid body model was created using the 3D coordinates of anatomical landmarks (head, trunk, upper arms, forearms, hands, pelvis, thighs, shanks and feet) obtained in the static standing position. The geometry of each segment in the model was calculated using Hanavan’s mathematical model for the human body (Hanavan 1964). The mass of each segment in the model was calculated using Dempster’s anthropometric data (Dempster and Gaughran 1967). Vertical GRF over 20 N was used to detect the instants of contact and take-off during the first step (Slawinski et al. 2010a). The analysis items were as follows:

1. *2*-*m sprint time* This was calculated using the displacement of the COM. The 2 m was the longest running distance using an integer within the capture length (3.0 m). This 2-m distance is used to assess performance in the first accelerated run (Otsuka et al. 2014).
2. *Step length to first step* Horizontal distance between the toe of the front leg at set position and the next toe of the opposite leg of first step was calculated.
3. *Step frequency to first step* Step frequency from start to touchdown of first step was calculated. Step frequency was calculated using the inverse of the stepping duration.
4. Variables during the first step: Increase in sprint speed was calculated by dividing the horizontal GRF impulse during the first step by the body mass. Sprint velocity at instant of take-off was calculated using the horizontal velocity of the COM. Forward leaning angle of the COM was calculated using the mean value of orientation angle of the vector from the center of pressure to the COM during the first step using the arc tangent. The vector was projected onto the sagittal plane. Contact time was calculated during the stance phase of first step using vertical GRF. Humerothoracic joint angles and ROMs were calculated using 3D Euler angle with creating local coordinate systems of thorax and humerus and joint coordinate system by the method of Wu et al. (2005) (Fig. 1c). X-axis rotation to be the most important because the arm-swing motion primarily involves flexion and extension motion of the humerothoracic joint (Slawinski et al. 2010b); therefore, the rotation sequence of the humerothoracic joint was X–Y–Z. The humerothoracic joint ROMs were calculated using the difference between the maximal and minimal values during the first step. Hip extension, knee flexion, ankle flexion and foot forward leaning angles of the stance leg were calculated using these angles in the sagittal plane two-dimensionally because the sprint performance is finally determined by the leg motion onto anterior direction.

### Statistical analysis

The average value of the all four trials was used in each condition for further analysis. All data are shown as mean ± SD. Prior to testing for mean differences, the samples were tested for normal distribution using the Kolmogorov–Smirnov test. In the case of normally distributed samples, paired t-tests were applied. Otherwise, the Wilcoxon test was used for paired samples. The effect size between free and constraint conditions was calculated for each variable using Cohen’s *d* (Cohen 1988). Relative magnitude of the effect was assessed based on Cohen’s guidelines with *d* ≤ 0.20 representing a small difference, >0.20 but <0.80 a moderate difference and ≥0.80 a large difference between the means. The Cohen’s d statistic was calculated when means and SD were reported. The statistical power was calculated using the G*Power software version 3.1.7 (Franz Faul, Keil, Germany). The significance level was set at *P* < 0.05.

## Results

In the free condition, the 2-m sprint time was significantly shorter than that in the constraint condition (Table 1). Step length to first step in free condition was significantly longer than that in constraint condition. No significant difference of step frequency to first step was observed between the free and constraint conditions. The increase in sprint speed, sprint speed at the instant of take-off of first step, and the forward leaning angle of COM during the first step were significantly greater than those in the constraint condition. There was no difference in the contact time during the first step between the two conditions. The effect size and statistical power of these variables are shown in Table 1.

**Table 1:** Sprint performance (mean ± SD)

	Condition		Cohen’s *d*	*Power*
	Free	Constraint		
2-m sprint time (s)	0.73 ± 0.04	0.76 ± 0.04*	0.75	0.707
Step length to first step (m)	0.88 ± 0.07	0.84 ± 0.07*	0.42	0.346
Step frequency to first step (Hz)	2.15 ± 0.18	2.16 ± 0.17	0.08	0.080
Variables during the first step				
Increase in sprint speed (m s^−1^)	0.94 ± 0.10	0.91 ± 0.08*	0.32	0.246
Sprint speed at instant of take-off (m s^−1^)	4.31 ± 0.20	4.19 ± 0.23*	0.55	0.490
Forward leaning angle of center of mass (°)	25.48 ± 1.99	24.49 ± 1.95*	0.52	0.430
Contact time (s)	0.186 ± 0.021	0.186 ± 0.010	0.02	0.057

* Significantly (*P* < 0.05) different from the free condition

**Fig. 2:**
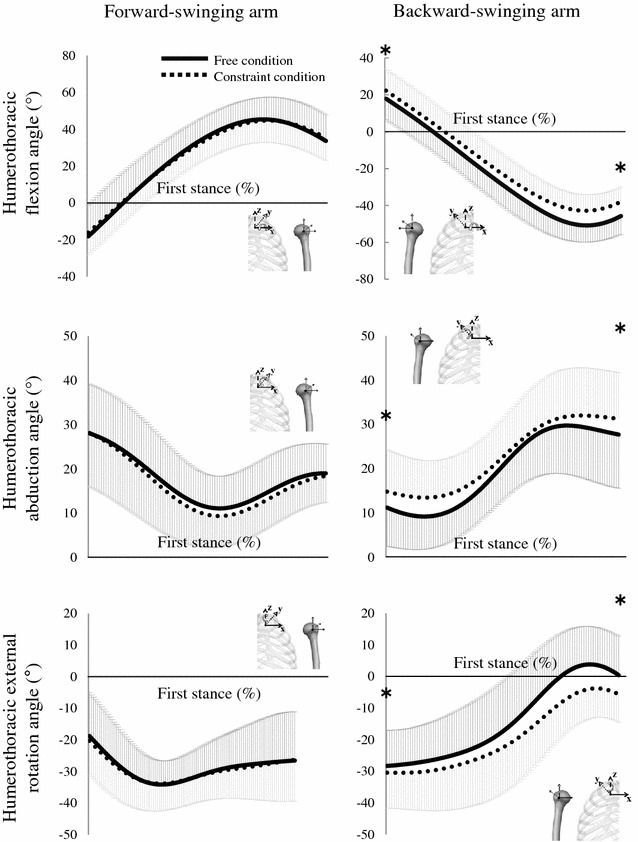
Mean ± SD of humerothoracic flexion, abduction and external rotation angles of the forward- and backward-swinging arms during the first step (*P < 0.05). *Solid lines* show these angles in free condition and *dotted line* shows those in constraint condition

With regard to the forward-swinging arm, the humerothoracic joint was flexed, adducted and internal rotated during the first step (Fig. 2). At the instants of foot-contact and take-off during the first step, no significant difference in the humerothoracic flexion, abduction and external rotation angles were seen between these two conditions. With regard to the backward-swinging arm, the humerothoracic joint was extended, abducted and external rotated during the first step. At the instants of foot-contact and take-off during the first step, the humerothoracic joint was significantly flexed, abducted and internal rotated in the constraint condition rather than the free condition. Whilst the ROM of humerothoracic extension in the constraint condition was significantly smaller than that in the free condition (65.8° ± 11.2° vs 69.4° ± 10.2°; *P* < 0.05; *d* = 0.34; *power* = 0.254), no significant difference in the other ROM of humerothoracic joint in forward- and back-ward swinging arms was seen between the two conditions.

**Fig. 3:**
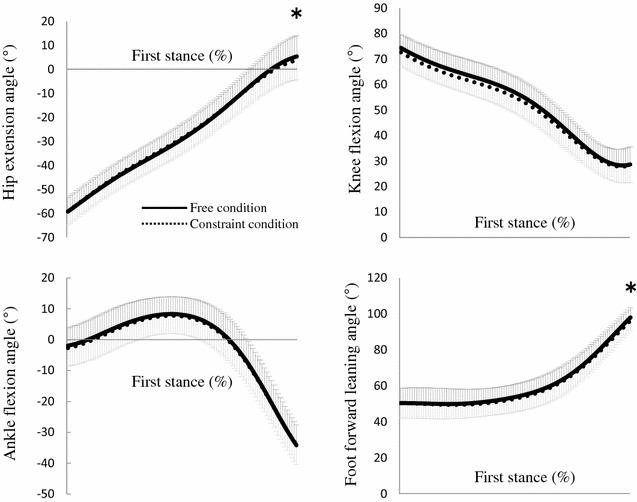
Mean ± SD of hip extension, knee flexion, ankle flexion and foot forward leaning angles of stance leg during the first step (*P < 0.05). *Solid lines* show these angles in free condition and *dotted line* shows those in constraint condition

The hip, knee and foot forward leaning angles were extended during the first step (Fig. 3a). The ankle angle was flexed during the first half of stance and was extended during the second half. At both the instants of foot contact and take-off during the first step, no significant difference in knee and ankle flexion angles was observed between the constraint and free conditions. Although no significant difference in hip extension and foot forward leaning angles was seen at the instant of foot contact during the first step between the two conditions, at the instant of take-off, the hip extension and foot forward leaning angles in the constraint condition were significantly smaller than those in the free condition.

## Discussion

The study was the first to clarify the changes in sprint speed and kinematic parameters in the constraint condition of the scapulothoracic joints. During the first step, the humerothoracic extension ROM of the backward-swinging arm and the hip extension angle of the stance leg at the instant of take-off in the constraint condition were significantly smaller than those in free condition even though the statistical powers were low. In the free condition, the 2-m sprint time was significantly shorter than that in the constraint condition. These findings suggest that in first accelerated running, the scapular behavior in the backward-swinging arm coordinates with humerothoracic extension and contralateral hip extension, thereby enhancing sprint speed.

In previous studies, many authors have shown interest in the relation between arm swing and sprint performance. Sprint speed was significantly different between the without constraint condition and with constraint condition created carrying a field hockey stick; the difference was significant at 1.2 % (Wdowski and Gittoes 2013). Compared with sprinting 20 m without the ball, that with the ball in both hands led to the greatest decrement in sprint speed, although left-arm and right-arm handed methods also reduced the sprint speed (Grant et al. 2003). These significant differences were 1.7, 1.0 and 0.9 %, respectively. In this study, the sprint time in the constraint condition was longer than that in the free condition. This relative difference between the two conditions was 4.1 % and was interestingly higher than that in previous studies (Grant et al. 2003; Wdowski and Gittoes 2013). Compared to the constraint of the arm-swing motion, the limited scapular motion may have a stronger effect on the decrease of sprint speed.

X-axis rotations of humerothoracic joints are important because the arm-swing motions primarily involve humerothoracic flexion and extension motions (Slawinski et al. 2010b). In the constraint condition compared with the free condition, the humerothoracic flexion ROM of the forward-swinging arm did not change in first accelerated running. In contrast, humerothoracic extension of the backward-swinging arm was smaller in the constraint condition compared with that in the free condition. Moreover, in the functional tests, the humerothoracic extension ROM was limited more than that of humerothoracic flexion (44.9 % decrease vs. 17.1 % decrease). These findings suggest that compared with the humerothoracic flexion motion, extension is more sensitive to constraint of the scapulothoracic joint. The coracohumeral ligament, which is a part of the hard tissue in front of the glenohumeral joint, has a greater effect on the stability of glenohumeral joint when performing the humerothoracic extension than during the flexion motion (Arai et al. 2014). The glenohumeral extension ROM is smaller than that of glenohumeral flexion because the coracohumeral ligament is strained during early glenohumeral extension (Izumi et al. 2011). Therefore, a larger 3D motion of the scapulothoracic joint is more required for sufficient humerothoracic extension than that for humerothoracic flexion. The difference in the anatomical features of the shoulder between the forward- and backward-swinging arms might lead to different contribution of the scapulothoracic and glenohumeral joints to the humerothoracic joint during the first step.

**Fig. 4:**
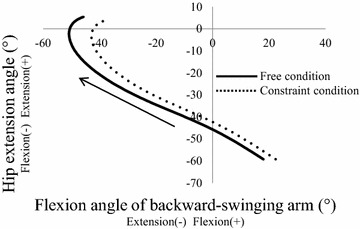
Relationship between the extension angle backward-swinging arm and the contralateral hip extension angle of the stance leg during the first step. *Solid lines* show these angles in free condition and *dotted line* shows those in constraint condition

By constraining scapular motion, the angle and ROM of humerothoracic extension of backward-swinging arm were decreased. The upper limbs play an important role in maintaining and adjusting balance by opposing the motion of the lower limbs (Mann 1981). The shoulder of the backward-swinging arm is contralateral to the hip joint of stance leg, and these are the most proximal joints in each limb. These may lead to a decrease in the extension angle of only the hip joint of the stance leg at the instant of take-off (Fig. 4). A previous cross-sectional study reported that hip extension velocity during the stance phase relates to the sprint speed in the 100-m race (Ito et al. 2008), suggesting that the large motion of hip extension during the stance phase is the key to enhancing sprint speed. Our results supported the finding of this previous study.

Whilst the hip extension angle of the stance leg at the instant of take-off in the free condition was larger than that in the constraint condition, no significant difference in knee and ankle angles of the stance leg was seen between the two conditions. This may be the cause of the lower foot forward leaning angle of the stance leg in the constraint condition than that in the free condition, and finally the lower forward leaning angle of the COM during the first step. Higher horizontal accelerations were generated by more forward leaning of the body at toe-off (Kugler and Janshen 2010). The forward leaning angle of the COM at the instant of take-off has a positive correlation with horizontal displacement of the COM during the stance phase (Hunter et al. 2004), suggesting that the forward leaning angle of the COM at take-off is important for enhancing sprint speed. Our results supported these findings of previous studies, and this limited leg motion of the stance leg in the constraint condition may cause loss of whole-body balance during kicking ground, thereby reduced sprint speed. Therefore, the 3D scapular behavior in first accelerated running may play a role in increasing humerothoracic extension of the backward arm and hip extension of the stance leg, thereby contributing to coordinated whole-body balance for great sprint acceleration. In addition, step length to first step was longer in the free condition than that in the constraint condition. This suggests that scapular behavior affects the beginning of motion in sprint running immediately before the first step, thereby affecting the ability of the sprinter to enhance sprint speed. Thus, our hypothesis was accepted.

There is a limitation in this study. Our study included a small number of participants, which might underpower our analysis of some variables. The findings in this study were based on ten participants; therefore, the statistical powers of some variables were low. Further study is needed with a larger sample size. Despite this limitation, our findings indicate an association between scapula behavior and fast sprinting in the first zone of accelerated running.

## Conclusions

When the arm-swing motion was restricted by constraining scapular motion, it clearly limited the humerothoracic extension of the backward-swinging arm. As a result, this scapular constraint affected the stance-leg motion and whole-body position during the first step, thereby reducing the sprint speed. Our results demonstrated that 3D scapular motion is related to the sprint speed in accelerated running. Our findings may be applied to the followings: for athletes with low scapular flexibility, improving it by performing scapular flexibility exercises is important as it may be related to improvement in the generated sprint speed. Also, for female athletes, the effects of compressing both scapulae by a sports bra would affect to sprinting performance in first accelerated running. In fact, a sport bra affect to running kinematics and the performance relative to without that (White et al. 2009). Further researches are required to clarify these questions in detail in the future.
